# Supplementary material for: Mixed Polymeric Micelles for Rapamycin Skin Delivery
Source: Pharmaceutics. 2022 Mar 4;14(3):569. doi: 10.3390/pharmaceutics14030569 (PMC8948846; doi:10.3390/pharmaceutics14030569)
Supplement: Supplementary file 1 [file pharmaceutics-14-00569-s001.zip › pharmaceutics-1604423-SM FINAL.pdf]

Supplementary Materials

Mixed Polymeric Micelles for Rapamycin Skin Delivery

Guillaume Le Guyader, Bernard Do, Ivo B. Rietveld, Pascale Coric, Serge Bouaziz, Jean-Michel Guigner, Philippe-Henri Secretan, Karine Andrieux and Muriel Paul

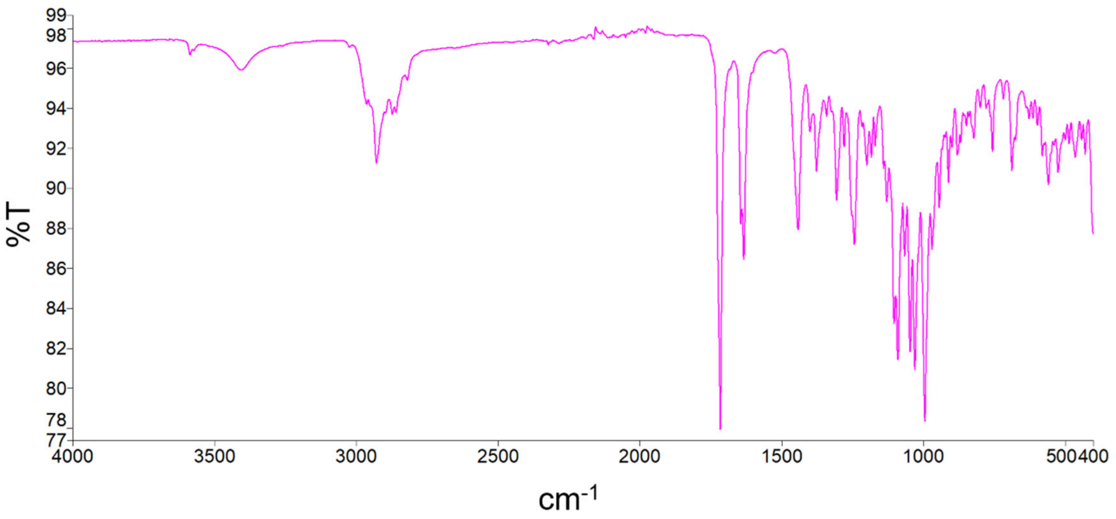

Figure S1. FTIR spectrum of rapamycin.
